# Liposarcoma: Advances in Cellular and Molecular Genetics Alterations and Corresponding Clinical Treatment

**DOI:** 10.7150/jca.36380

**Published:** 2020-01-01

**Authors:** Lingge Yang, Shiqi Chen, Peng Luo, Wangjun Yan, Chunmeng Wang

**Affiliations:** 1 Department of Musculoskeletal Oncology, Fudan University Shanghai Cancer Center, Shanghai, China; 2 Department of Oncology, Shanghai Medical College, Fudan University, Shanghai, China

**Keywords:** Liposarcoma, Cytogenetics, Molecular genetics, Epigenetics, Clinical Treatment

## Abstract

Liposarcoma is a malignant tumor of mesenchymal origin with significant tissue diversity. It is composed of adipocytes with different degrees of differentiation and different degrees of heteromorphosis. It is not sensitive to traditional radiotherapy and chemotherapy and has a poor prognosis. In recent years, with the rapid development of basic immunology, molecular genetics and tumor molecular biology, the histological classification of liposarcoma has become increasingly clear. More and more new methods and technologies, such as gene expression profile analysis, the whole genome sequencing, miRNA expression profile analysis and RNA sequencing, have been successfully applied to liposarcoma, bringing about a deeper understanding of gene expression changes and molecular pathogenic mechanisms in the occurrence and development of liposarcoma. This study reviews the present research status and progress of cellular and molecular alterations of liposarcoma and corresponding clinical treatment progress.

## Introduction

Liposarcoma is a common type of soft tissue sarcoma that accounts for about 20% 1 of all adult sarcomas. Liposarcoma often develops in deep soft tissues of lower limbs and retroperitoneal parts, accounting for 24% and 45% 2 of limb sarcomas and retroperitoneal soft tissue sarcomas respectively. In accordance with the typical morphological characteristics and biochemical characteristics showed at different stages of adipocyte differentiation, liposarcoma is divided into 3 groups and 5 types by WHO: well-differentiated/dedifferentiated liposarcoma (WDL/DDL), myxoid/round-cell liposarcoma (MRCL) and pleomorphic liposarcoma (PLS) 3. Such kind of classification tries to determine different subtypes of liposarcoma according to their clinical features, morphological features, immunophenotypic and genetic characteristics, etc., making liposarcoma become an independent disease species to facilitate research and comparison of clinical, pathological, genetic data and molecular pathogenesis. This study reviews the cellular and molecular genetics alterations and corresponding clinical treatment of liposarcoma.

## Well-differentiated/Dedifferentiated Liposarcoma (WDL/DDL)

WDL/DDL is most common type of liposarcoma, accounting for about 40% to 45% 4 of liposarcoma. Superficial WDL is also called atypical lipomatous tumor (ALT). Histologically, WDL consists mainly of mature adipocytes, atypical stromal cells, and a small number of scattered fat mother cells, which is quite similar to normal adipose tissue and mature benign lipoma tissues. Cytogenetic studies have showed that WDL/DDL is characterized by supernumerary ring chromosome and/or giant marker chromosome composed of amplified products from the q13-15 region on Chromosome No. 12 5. Many genes, such as *MDM2, CDK4, HMGA2, CPM, SAS/TSPAN31, DYRK2, YEATS4* and others, amplify along with WDL/DDL 6.

Studies have showed that gene amplification exists in the 12q12-21 and 10p11-14 regions of the WDL/DDL cell lines 7. Among them, *MDM2* and *CDK4* keep being amplified and expressed. Both genes are proto-oncogenes, and the encoded proteins are involved in the regulation of cell cycles. *MDM2* expression products are also transcriptional activation inhibitors of p53, which inhibit the transcription of p53 and lead to cell proliferation 4, 5, 7-11. H*MGA2* expression products can regulate transcription through DNA structural modification and cross-linking with other enhanceosome proteins, which are normally expressed during embryonic development instead of normal somatic cells. However, for WDL/DDL, *MDM2* amplification is accompanied by *HMGA2* dysregulation, presenting its oncogenic property 8. *CPM* is at the downstream location of *MDM2* and the encoded protein is associated with many functions, such as adipose tissue differentiation, osteogenic differentiation, inflammation, and coagulation 9. *FRS2* expression products can be activated into fibroblast growth factor receptor (FGFR) signal, and the abnormal activation of this signal can lead to tumor formation, tumor angiogenesis, and metastasis 10, 11. A study found that *FRS2* was amplified in 93.2% (132/146) of WDL/DDL, and the FRS2/CEP12 ratio in DDL was significantly higher than that in WDL (P = 0.0005) 12.

WDL is a well-differentiated type of liposarcoma with comparatively weak invasive ability basically without metastasis. However, WDL can be dedifferentiated and be converted to DDL, so as to obtain stronger invasive ability with potential local recurrence and distant metastasis. This dedifferentiation occurs in approximately 10% of WDL 13. DDL morphologically consists of WDL region and a suddenly-transitioned region of non-adipose tissue sarcoma. Thus, it is not difficult to understand that DDL has the same cellular and molecular genetic characteristics as WDL.

The difference is that DDL has additional genetic changes, especially co-amplification of genes in chromosome 6q23 and 1p32 regions, such as *JUN* and ASK1/MAP3K5 5, 7, 13-16. Both of the two gene-encoded products can participate in the conduction of c-Jun N-terminal protein kinase (JNK) signal pathway. *JUN* expression products can regulate the activity of factors involved in adipocyte transcription, and *ASK1/MAP3K5* can encode the kinase in the upstream location of *JUN*. The amplification of *JUN* or *ASK1/MAP3K5* is related to the fact that WDL dedifferentiation leads to the tissue type changing to DDL 15. However, in a phase II clinical trial, plitidepsin, which activated the JUN pathway to induce apoptosis, did not show ideal clinical effect in treating patients with advanced DDL 16. The systematic connection of the amplification of both genes and DDL occurrence has not been fully confirmed.

In addition, *DDIT3, PTPRQ, YAP1*, and *C/EBPα* also have different degrees of amplification. The copy number and mRNA levels of these four genes are correlated with the expression level of *JUN* 17. The transcription factor, C/EBPα, which is involved in cell cycle regulation and cell differentiation, is studied more frequently. Its lack of expression is an important factor for DDL to maintain differentiation and inhibit apoptosis 18. Besides, the expression of anti-aging protein Klotho down-regulates in DDL compared with WDL and adipose tissue, which is related to poor prognosis; in addition, it can regulate the drug sensitivity of thapsigargin and gemcitabine by inhibiting ERK1/2 signal transduction, which provides a new therapeutic strategy for DDL 19.

### Myxoid and Round-Cell Liposarcoma (MRCL)

MRCL is the second largest category of liposarcoma. It is usually composed of round cell liposarcoma (RCL) and myxoid liposarcoma (MLS) in histomorphology. In general, RCL is more invasive than MLS and the higher the proportion of the former is, the worse the prognosis is indicated 20. The most prominent cytogenetic feature of MRCL is that about 95% 21 of cases have specific t(12;16)(q13;p11) chromosomal translocation, which produces FUS-DDIT3 fusion protein (also known as TLS-CHOP fusion protein), while about 5% 22 of cases have t(12;22) (q13;q12) chromosomal translocation, producing EWSR1-DDIT3 fusion protein.

FUS-DDIT3 and EWSR1-DDIT3 show a high degree of specificity and can be used as characteristic diagnostic indicator. These fusion proteins are important molecules for development of sarcoma and inhibition of adipogenesis and play crucial roles in the pathogenesis of MRCL. The expression level of FUS-DDIT3 fusion protein is also positively correlated with cell differentiation 23. In addition, studies have shown that the FUS-DDIT3 fusion gene can enhance the invasion ability of MRCL by activating the SRC/FAK/RHO/ROCK signal axis, and the expression level of FAK is related to the degree of malignancy and the tumor grade 24.

Interestingly, for most of MRCL, *TP53* is not mutated and can produce functionally normal p53, and once this gene is mutated, the invasive ability of MRCL will also be strengthened 25.

It has been shown that MRCL can cause gene mutation of *EGFR, PDGFRB, RET, MET* and *VEGFR1* through the interaction of the autocrine/paracrine loop and the receptor tyrosine kinase (RTK), and it can keep activating the signaling pathway of the downstream PI3K/Akt, leading to the over-expression of growth factor receptor RET and IGF1R, which is related to the transformation of MLS to RCL, increasing invasiveness, and poor prognosis 20, 26-28.

Round cell took up more than 5% of MRCL, indicating a poor prognosis 29. In order to further investigate the process of MLS transforming into RCL, Cecco et al. 30 used gene expression profiling, immunohistochemistry, biochemical analysis, and other techniques to study two groups of samples containing only MLS and RCL, respectively, and found that in this process, the silence of the *DLK1-DIO3* genomic region at 14q32 resulted in the over-expression of genes such as *YY1/C-MYC/HDAC2* that promoted rapid cell cycle progression. And *MKNK2, MSX1* and *TRIM71* encoded for cell proliferation and stem cell formation, were also over-expressed. MLS developed into RCL by crossing epigenetic silencing restriction point, rearranging its stem cell sample differentiation markers.

Nezu et al. 31 used miRNA microarray analysis to explore the transformation process and found that miR-135b was expressed at a higher level in RCL, which could be used as an oncogenic miRNA. Through the miR-135b/THBS2/MMP2 axis, miR-135b strengthened the ability of MLS to grow, invade and metastasize. At the same time, the density of MLS cells increased and the extracellular collagen matrix decreased, resulting in a change in the histopathology of MLS and eventually transformation to RCL.

## Polymorphic Liposarcoma (PLS)

PLS accounts for less than 5% 32 of liposarcoma, which is the rarest type. With high local recurrence rate and distant metastasis rate, PLS is more invasive than other types of liposarcoma, and is less sensitive to conventional treatment. Histologically, PLS is composed of many irregular cell groups and abundant isolated, non-adherent cells and has distinct polymorphism and common characteristic sheets of bizarre pleomorphic mono or multivacuolated adipoblasts 33-35.

The cytogenetic feature of PLS is complex aneuploid karyotypes with complex genomic amplification and deletions 34, 36. Barretina et al. 37 found mutations in genes such as *TP53, RB1 and NF1* in PLS by DNA sequencing, while Ghadimi et al. 36 found many biomarkers, such as peroxisome proliferator-activated receptor gamma (PPAR-γ), VEGF, survivin protein, B-cell leukemia 2 and matrix metalloproteinase 2, were over-expressed in PLS; in addition, they observed the presence of high-frequency deletion of retinoblastoma protein and high-frequency gene mutation of *TP53* (about 60%) in PLS.

In general, although many chromosome structures and gene expression abnormalities have been discovered in PLS, no characteristic or constant chromosomal aberrations or molecular alterations have been found.

## The Research Significance

### Diagnosis and Differential Diagnosis

In WDL/DDL, continuous amplification of *CPM* can be detected in all tissues and can be used as a new indicator for diagnosis 9. The cancer testis antigen NY-ESO-1 seems to be a useful immunohistochemical marker to support the diagnosis of MRCL because of its high sensitivity and specificity 38.

In terms of differential diagnosis, it is difficult to diagnose only from the histomorphological manifestations of WDL and DDL. The current diagnostic method is guided by MDM2 and CDK4 immunohistochemistry and determined by the amplification of the corresponding genes. Recently, p16 immunohistochemistry has been considered to be an effective diagnostic biomarker. A study has shown that 68% of WDL and 72% of DDL could express the above three proteins, while all WDL and 93% of DDL expressed at least two of them. Through performing MDM2 immunohistochemical test on both, they found that the sensitivity and specificity were 86% and 74%, while CDK4 was detected to be 86% and 89%, p16 was detected to be 93% and 92%, and the the sensitivity and specificity was 71% and 98% when they combined detection of the three markers 39. In conclusion, the study suggested that MDM2, CDK4, and p16 immunohistochemical detection were effective supplementary means of identifying WDL and DDL from other types of liposarcoma.

P16 is gene-encoded by *CDKN2A* and inhibits the progression of the cell cycle by binding to CDK4, which is the most sensitive and specific marker for detecting WDL/DDL. However, Kang et al. 40 believed that when distinguishing retroperitoneal DDL from other common retroperitoneal tumors, especially leiomyosarcoma and desmoid tumors, p16 is not as practical as MDM2 and CDK4 due to its low specificity.

Some researchers further analyzed the three proteins above and found that MDM2+/p16+ were all WDL, MDM2-/p16- was benign fatty tumor, MDM2/CDK4/p16 was DDL, and MDM2-/CDK4-/p16- was a kind of undifferentiated sarcoma. The results showed that the combination of three proteins test could effectively identify WDL and DDL 41.

### To Find Suitable Therapeutic Targets

Clinically, the curative management for localized disease is surgical resection, combined with or without radiotherapy. Systemic treatment with chemotherapy and molecular targeted agents is one of the main therapeutic modalities in patients with advanced or metastatic disease. With increasing number of studies in molecular basis of pathogenesis and emerging new therapeutic targets, the treatment outcome of liposarcoma will be greatly improved in the future.

In WDL/DDL, CDK4 is continuously amplified in about 90% of cases, and a CDK4/6 inhibitor, palbociclib, showed certain efficacy in the treatment of advanced CDK4+ WDL/DDL, for the 66% progression-free survival (PFS) rate at 12 weeks, and the median PFS was 18 weeks 42. Subsequent researches have also been carried out gradually. Some researchers found that palbociclib combined with recombinant methioninase had excellent anti-tumor activity in the doxorubicin-resistant patient-derived orthotopic xenograft animal model 43. A study reported that the therapeutic effect of CDK4/6 inhibitors on MDL/DDL needed to be achieved by down-regulating the expression of MDM2 protein by PDLIM7 and CDH18 44.

The clinical study of MDM2 as a therapeutic target for WDL/DDL was reported in 2012. Ray-Coquard et al. 45 used MDM2 antagonist (RG7112) to treat 20 patients, of which one patient got complete response (CR) and 14 patients got stable disease (SD), showing a certain clinical efficacy. However, the adverse reactions were serious, which limited its clinical application. Subsequently, MDM2 inhibitors were also used in clinical trials. A phase I clinical trial of ALRN-6924 in the treatment of advanced solid tumors has achieved some outcomes according to its preliminary report, of which one patient with liposarcoma received partially responsive (PR) 46. Similarly, one patient with DDL received PR in a phase I clinical trial of DS-3032b for WDL/DDL 47. According to the results, compared with RG7112, the latter two had fewer adverse reactions and were more suitable for clinical use. Further detailed reports and clinical trials are worth looking forward to.

It is worth mentioning that although both MDM2 and CDK4 have corresponding targeted inhibitors for clinical trials, *in vitro* experiments have shown that the cytotoxicity of the two drugs against sarcoma cell lines is mutually antagonistic 48. Therefore, careful consideration must be given to the combination of CDK4 and MDM2 inhibitors to treat WDL/DDL.

In DDL, many other potential therapeutic targets have been reported. FRS2 is often amplified, and its encoded protein is not normally present in normal fat or preadipocytes, so FRS2 may be an effective target for this type of liposarcoma 10, 11. *In vitro* and *in vivo* experiments have shown that pan-FGFR inhibitor LY2874455 has clinical value in the treatment of DDL for FRS2 amplification 49. In addition, methylation of C/EBPα is found in 24% of DDL while demethylation pharmacotherapy can restore the expression of C/EBPα in DDL cells so as to inhibit the proliferation of DDL cells *in vitro* experiment as well as promote apoptosis and slow down the tumor growth *in vivo* 50. It suggests that demethylating agent may be a potential therapeutic agent for DDL. Some researchers also found that *STAT6* was located in 12q13 and was amplified in about 11% of DDL 51. The encoded product was STAT6, a member of the STAT family, which was cytoplasmic transcription factor. The over-expression of this transcription factor was closely related to tumor growth and its inhibitor was of potential therapeutic value to this type of DDL.

Immune checkpoint inhibitors have opened up a new way for the treatment of DDL. Studies have shown that PD-L1 is highly expressed in DDL cell lines, and patients with the PD-L1 expression ≥1% have a significant decrease in recurrence-free survival (P = 0.027) and overall survival (P = 0.017). They also found that PD-L1 expression is mediated by IFN-γ, suggesting the possibility of combined treatment for DDL 52. A phase II clinical trial of pembrolizumab in the treatment of advanced sarcoma has been reported. Of ten patients with DDL enrolled, two cases received PR, four received stable disease (SD), median PFS was 25 weeks, and PFS rate was 60% at 12 weeks, which has clinical application value 53.

In MRCL, Pollack et al. 54 used immunohistochemistry and qPCR to detect 25 samples and found NY-ESO-1 expression in all their samples. They also found that the sensitivity of MRCL cell lines to antigen-specific lysis was demonstrated by using NY-ESO-1 specific, CD8+ T-cells, suggesting that this antigen should be used as a potential therapeutic target for MRCL. Subsequently, they reviewed the efficacy of CMB305, a therapeutic vaccine targeting NY-ESO-1, indicating that the vaccine can significantly improve the OS of MRCL patients 55. Hemminger et al. 56 also got similar conclusions and they found that *CTAG1B* and its mRNA were highly over-expressed in MRCL. These proteins also have potential value for targeted immunotherapy.

In addition to NY-ESO-1, other biomarkers also can be used as therapeutic targets for MRCL. Experiments showed that IGF-IR inhibitors could suppress the growth of MRCL cell lines, and IGF-IR/PI3K/Akt signaling pathway could be used as a specific therapeutic target of MRCL 26. HSP90 inhibitor could block the phosphorylation of ERBB3 and RET in MRCL, leading to tumor tissue necrosis, which was confirmed *in vitro* and *in vivo* experiments, thus HSP90 inhibitor was expected to become a medicine for treating MRCL 27. In MRCL tissue, CD68+ macrophage infiltration indicated a poor prognosis, and the possible mechanism could be that the secretion of heparin-binding EGF-like growth factor (HB-EGF) combines EGFR, resulting in a stronger invasive ability of MRCL invasion 28. Therefore, HB-EGF and EGFR also have the potential to be therapeutic targets of MRCL.

It has been reported in a case that using apatinib, a small molecule inhibitor of receptor protein tyrosine kinase, targeting VEGFR-1, VEGFR-2, PDGFRB, etc., for the treatment of advanced RCL, the patient achieved PR 57. There was also a case report showed that a patient with advanced PLS who had failed multiple chemotherapy regimens previously, achieved 3-month PFS and a high quality of life after received apatinib. Apatinib ehxibits a certain clinical efficacy in the treatment of liposarcoma 58.

The ongoing and upcoming clinical trials of targeted therapy and immunotherapy for liposarcoma are detailed in Table 1.

### Efficacy Assessment and Prognosis Prediction

In recent years, with the extensive application of RNA library depth sequencing technology and DNA microarray technology, miRNA has become a hot spot for studying the progress and prognosis of liposarcoma. The core role of miR-143 in the occurrence and development of WDL/DDL has been confirmed 59. Ugras et al. 60 found that miR-143 was highly expressed in normal adipocytes, while the expression was down-regulated in WDL, and if the expression was further down-regulated, the WDL could develop to DDL; in addition, recovery of miR-143 expression in DDL could inhibit its proliferation and induce apoptosis. Similarly, over-expression of miRNA-133a could inhibit the proliferation and regulate mitochondrial function and glycolysis ability of DDL cell lines. However, *in vivo* experiments showed that exogenous recombinant miRNA-133a had only the ability to regulate cell metabolism, and without proliferation inhibition 61. Recently, Mazzu et al. 62 has found that miR-193b regulates multiple oncogenic signaling pathways (such as PDGFR, TGF, and Wnt) by targeting PDGFR-β, SMAD4 and YAP1 proteins *in vitro*, and thus miR-193b plays a tumor-suppressing role in the WDL/DDL cell lines.

Borjigin et al. 63 found that the expression of miR-486 could be inhibited by the specific fusion protein FUS-DDIT3 in MRCL, while the addition of exogenous miR-486 could inhibit the growth of MRCL cells. Similarly, miR-145 and miR-451, members of miRNA with tumor-suppressing function, could inhibit the proliferation and differentiation of all types of liposarcoma and induce apoptosis by their over-expression *in vitro* 64. The aforementioned miRNA with tumor-suppressing function are good indicators of efficacy evaluation and prognosis, as well as potential therapeutic targets.

Zhang et al. 65 first reported that miR-155 had the important function of carcinogenesis in WDL/DDL, and miR-155 could strengthen the conduction of β-catenin signaling pathway through direct control of casein kinase 1α (CK1α), and increased the expression of cyclin D1, thus leading to the proliferation of DDL cell lines and accelerating cell cycle progression. Therefore, miR-155 could be a predictor of efficacy and prognosis of WDL/DDL, which was also further confirmed in other researchers' studies 66, 67.

Lee et al. 68 found the over-expression of miR-26a-2 was significantly associated with poor prognosis of patients with WDL/DDL and MRCL (P<0.05 in WDL/DDL group, P<0.001 in MRCL group). Exosomes-derived miR-25-3p and miR-92a-3p were found in liposarcoma, which could accelerate the proliferation, invasion, and metastasis of liposarcoma by stimulating the secretion of pro-inflammatory cytokine IL-6; in addition, these miRNAs could effectively distinguish patients with liposarcoma from healthy individuals and had the possibility of becoming a new non-invasive biomarker, so as to be used for early diagnosis of liposarcoma, evaluation of efficacy and prognosis 69.

## Conclusions

In the past decade, an in-depth research on the cellular and molecular pathogenesis of liposarcoma has brought about new ideas and methods for clinical diagnosis, treatment and prognosis. Although we are still at the early stage of translating these studies of cellular and molecular level into clinical applications, more and more new methods and technologies have been proposed and applied, which brings hope to patients and medical personnels. The advent of the era of precision medicine calls for further exploration of the pathogenesis of liposarcoma from cellular and molecular level so as to provide patients with individualized and accurate treatment.

## Figures and Tables

**Table 1:** Ongoing and Upcoming Clinical Trials of Targeted Therapy and Immunotherapy for Liposarcoma

Drug Name/Code	Targets	Pathological subtypes of liposarcoma	Recruitment	Phase	ClinicalTrials. gov ID
APX005M	CD40	Well/Dedifferentiated liposarcoma	Not yet recruiting	II	NCT03719430
Ribociclib/LEE011	CDK4/6	All	Recruiting	Ib	NCT03009201
Abemaciclib	CDK4/6	Dedifferentiated liposarcoma	Recruiting	II	NCT02846987
Ribociclib/LEE011	CDK4/6	Well/Dedifferentiated liposarcoma	Recruiting	II	NCT03096912
Ribociclib/LEE011+Everolimus	CDK4/6+mTOR	Dedifferentiated liposarcoma	Recruiting	II	NCT03114527
Regorafenib	c-Kit, B-Raf, Raf-1, RET, VEGFR1-3, PDGFR β etc.	All	Recruiting	II	NCT02048371
Sitravatinib/MGCD516	c-Kit, PDGFR α-β, c-Met, Axl etc.	Well/Dedifferentiated liposarcoma	Recruiting	II	NCT02978859
Selinexor/KPT-330	CRM1	Dedifferentiated liposarcoma	Recruiting	II/III	NCT02606461
Selinexor/KPT-330+Ixazomib	CRM1+20S proteasome	Dedifferentiated liposarcoma	Not yet recruiting	I	NCT03880123
Itacitinib/INCB39110	Jak1	Myxoid/round cell liposarcoma	Not yet recruiting	I	NCT03670069
MAGE-A4ᶜ¹º³²T cells	MAGE-A4	Myxoid/round cell liposarcoma	Recruiting	I	NCT03132922
HDM201+Ribociclib/LEE011	MDM2+CDK4/6	Well/Dedifferentiated liposarcoma	Active, not recruiting	Ib/II	NCT02343172
CD8+ NY-ESO-1-Specific T Cells+LV305±CMB305	NY-ESO-1	Myxoid liposarcoma	Recruiting	I	NCT03450122
NYCE T Cells	NY-ESO-1	Myxoid/round cell liposarcoma	Recruiting	I	NCT03399448
CMB305±Atezolizumab	NY-ESO-1±PD-L1	Myxoid/round cell liposarcoma	Active, not recruiting	II	NCT02609984
NY-ESO-1ᶜ²⁵⁹T cells	NY-ESO-1	Myxoid/round cell liposarcoma	Recruiting	II	NCT02992743
Pembrolizumab	PD-1	All	Not yet recruiting	II	NCT03899805
Pembrolizumab	PD-1	Myxoid/round cell liposarcoma	Recruiting	II	NCT03063632
Nivolumab+Nab-rapamycin	PD-1+mTOR	All	Recruiting	Ib	NCT03190174
Nivolumab±Ipilimumab	PD-1±CTLA-4	Dedifferentiated liposarcoma of the retroperitoneum	Recruiting	II	NCT03307616
Olaratumab	PDGFR α	All	Active, not recruiting	III	NCT02451943
Olaratumab	PDGFR α	Myxoid/round cell, pleomorphic or dedifferentiated liposarcoma	Recruiting	II	NCT02584309
Efatutazone	PPAR-γ	Myxoid liposarcoma	Active, not recruiting	II	NCT02249949
Pazopanib	VEGFR 1-3, c-Kit & PDGF-R	All	Recruiting	II	NCT01532687
Pazopanib	VEGFR 1-3, c-Kit & PDGF-R	Dedifferentiated, or myxoid liposarcoma	Recruiting	II	NCT02357810
Lenvatinib	VEGFR 2/3	Dedifferentiated, myxoid, or pleomorphic liposarcoma	Recruiting	Ib/II	NCT03526679

(The data come from https://www.clinicaltrials.gov and the latest update date is Mar 2, 2019) **Abbreviations:** ID: Identification; CDK: Cyclin-dependent kinase; mTOR: The mammalian target of rapamycin; RET: Rearranged during transfection; VEGFR: Vascular endothelial growth factor receptor; PDGFR: Platelet-derived growth factor receptor; MAGE-A4: Melanoma-associated antigen 4; NY-ESO: New York oesophageal squamous cell carcinoma; NYCE: NY-ESO-1-redirected CRISPR (TCRendo and PD1) Edited; PD-L1: Programmed death-ligand 1; PD-1: Programmed cell death protein 1; CTLA-4: Cytotoxic T lymphocyte-associated antigen-4; PPAR: Peroxisome proliferators-activated receptors.
